# Supplementary material for: Chemogenomic profiling of breast cancer patient-derived xenografts reveals targetable vulnerabilities for difficult-to-treat tumors
Source: Commun Biol. 2020 Jun 16;3:310. doi: 10.1038/s42003-020-1042-x (PMC7298048; doi:10.1038/s42003-020-1042-x)
Supplement: Supplementary file 1 — Supplementary Information [file 42003_2020_1042_MOESM1_ESM.pdf]

Supplemental Information

Chemogenomic profiling of breast cancer patient-derived xenografts reveals targetable vulnerabilities for difficult-to-treat tumors

Paul Savage, Alain Pacis, Hellen Kuasne, Leah Liu, Daniel Lai, Adrian Wan, Matthew Dankner, Constanza Martinez-Ramirez, Valentina Muñoz-Ramos, Virginie Pilon, Anie Monast, Hong Zhao, Margarita Souleimanova, Matthew G Annis, Adriana Aguilar-Mahecha, Josiane Lafleur, Nicholas R Bertos, Jamil Asselah, Nathaniel Bouganim, Kevin Petrecca, Peter M Siegel, Atilla Omeroglu, Sohrab P Shah, Samuel Aparicio, Mark Basik, Sarkis Meterissian, Morag Park

Supplementary Figures

Supplementary Figure 1

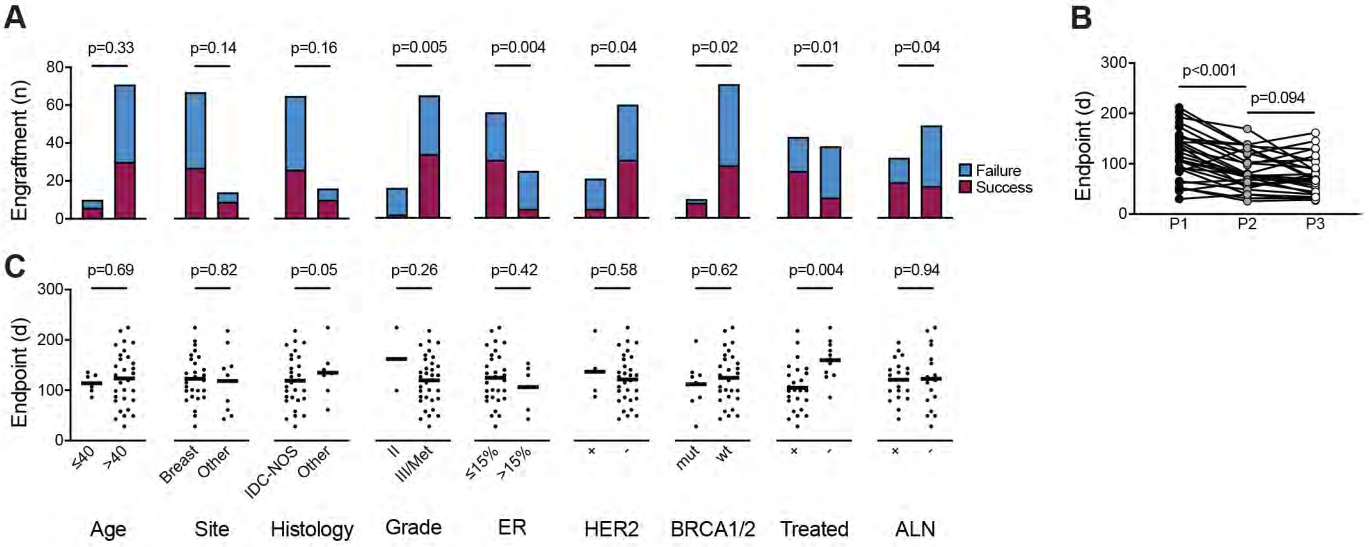

Supplementary Figure 1. Clinicopathologic features associated with PDX engraftment and growth kinetics. **A.** Stacked bar chart of engraftment success for various clinicopathologic factors. ER, estrogen receptor; ALN, axillary lymph node metastasis. **B.** Connected line plot for PDX growth kinetics (mean time for tumor diameter to reach 10 mm) over passages 1, 2 and 3 (P1-3; n=28, models with insufficient P1-3 data not included). **C.** Dot plot of PDX growth kinetics for various clinicopathologic factors. Line represents mean (n=36).

Supplementary Figure 2

A

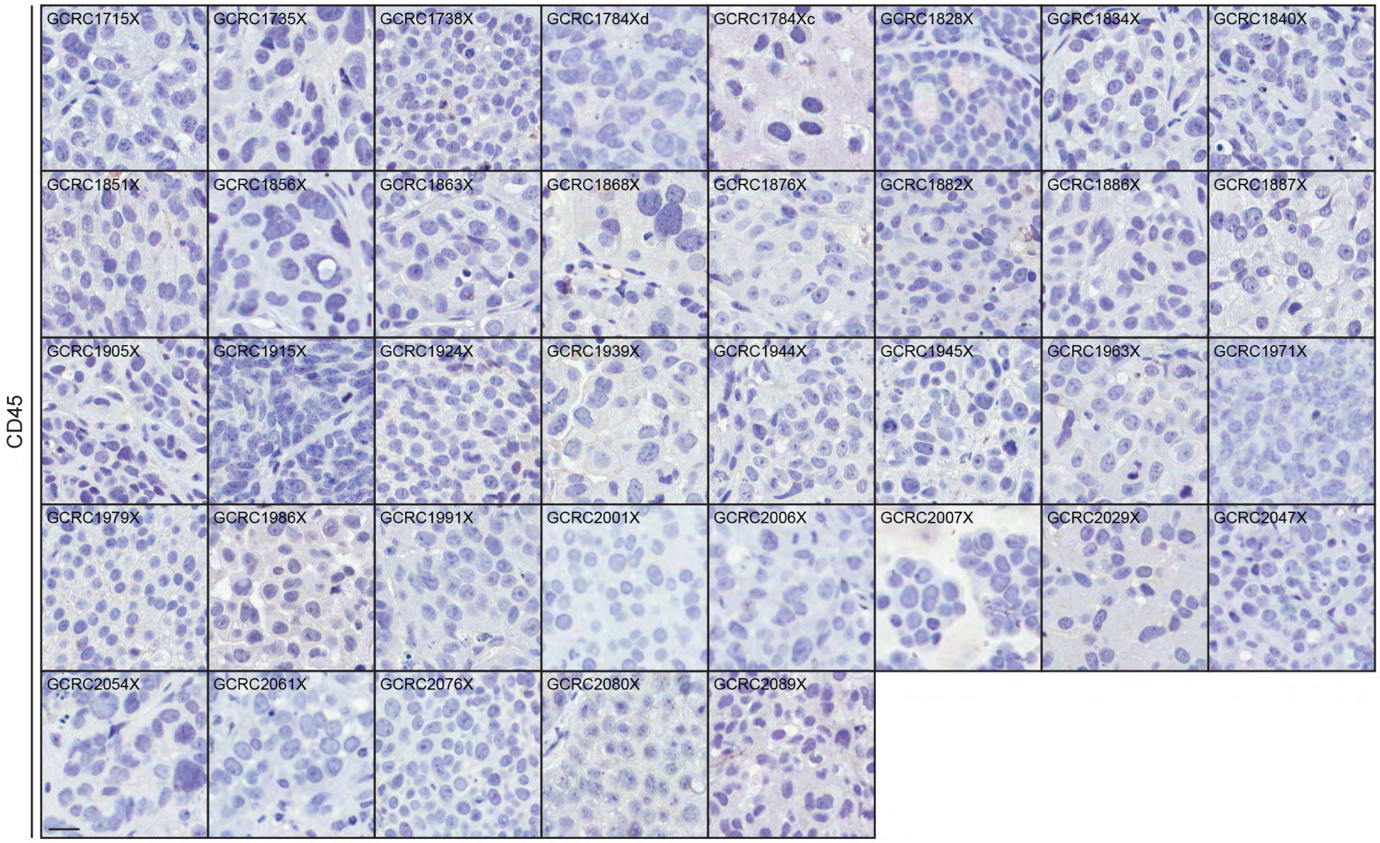

B

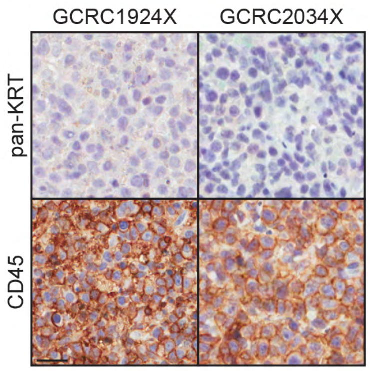

Supplementary Figure 2. PDX tumors represent human epithelial tumors. **A.** Representative immunohistochemical images of PDXs stained for CD45. Scale bar 20 µm. **B.** Representative immunohistochemical images of PDXs staining positive for CD45 and negative for pan-cytokeratin (pan-KRT). A separate P1 mouse of the GCRC1924X PDX line developed as a pan-KRT+/CD45- outgrowth which was used for serial propagation. All P1 mice for GCRC2034X contained pan-KRT-/CD45+ lymphoproliferative outgrowths, so the line was not included for further analysis. Scale bar 20 µm.

Supplementary Figure 3

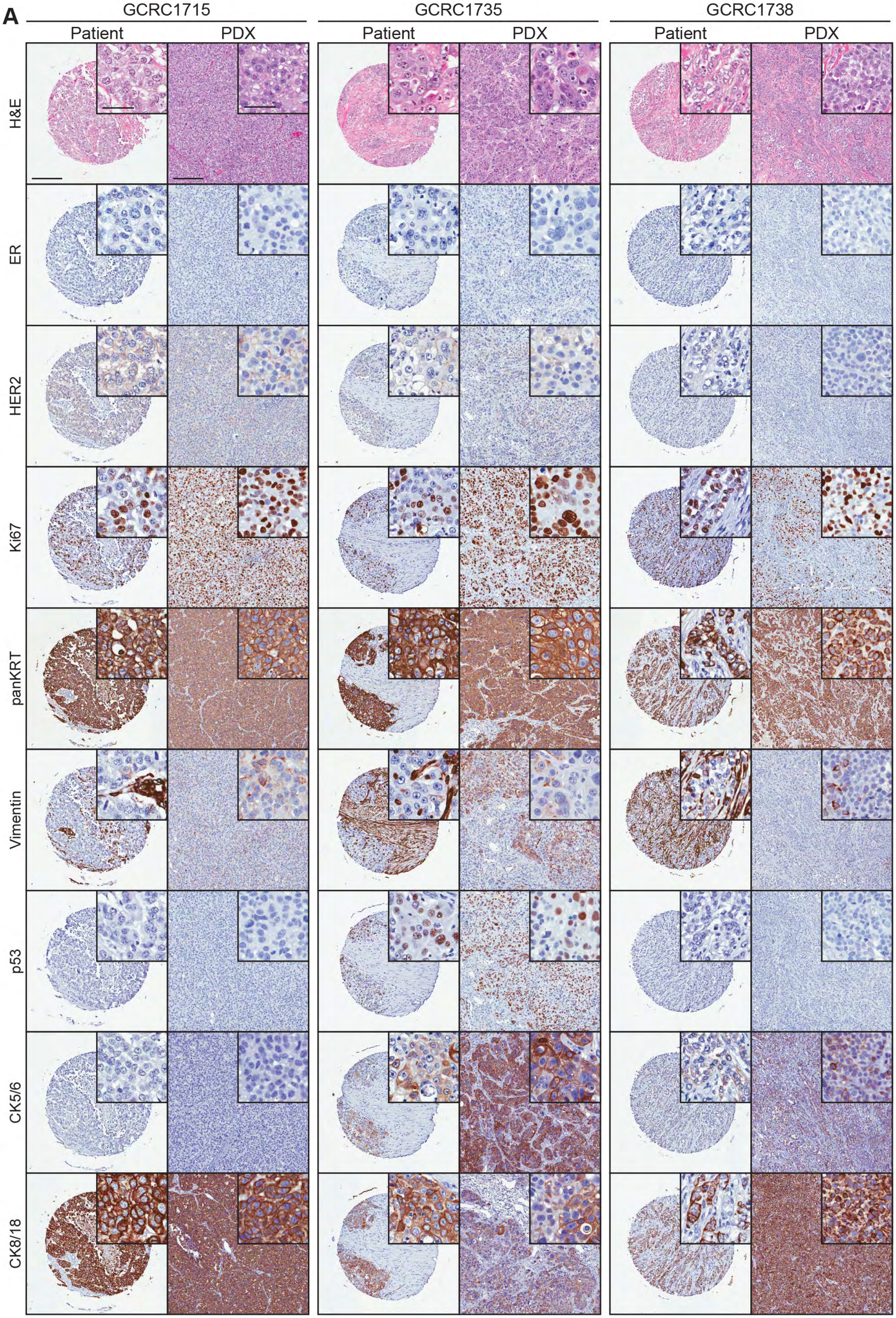

Supplementary Figure 3 (cont)

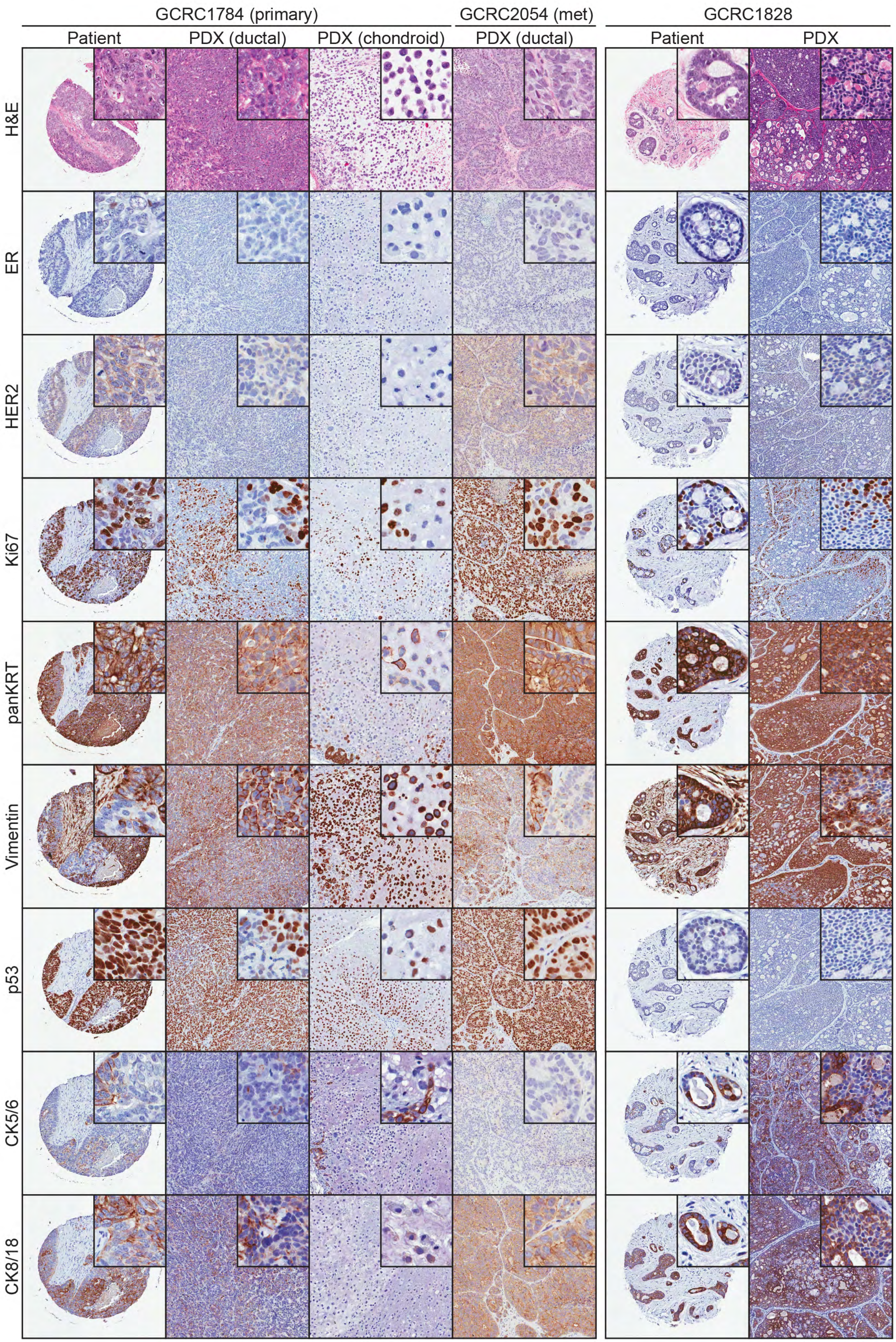

Supplementary Figure 3 (cont)

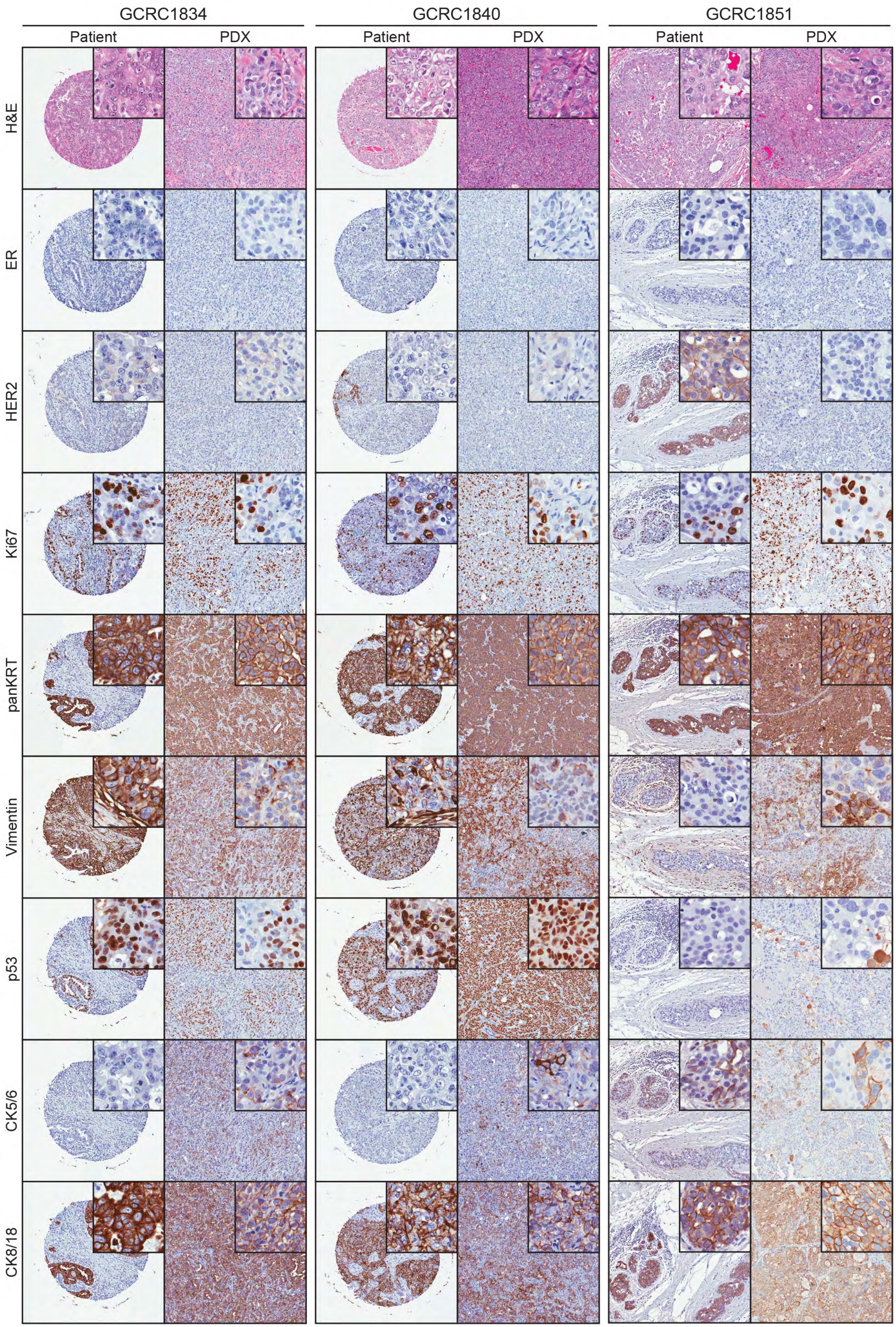

Supplementary Figure 3 (cont)

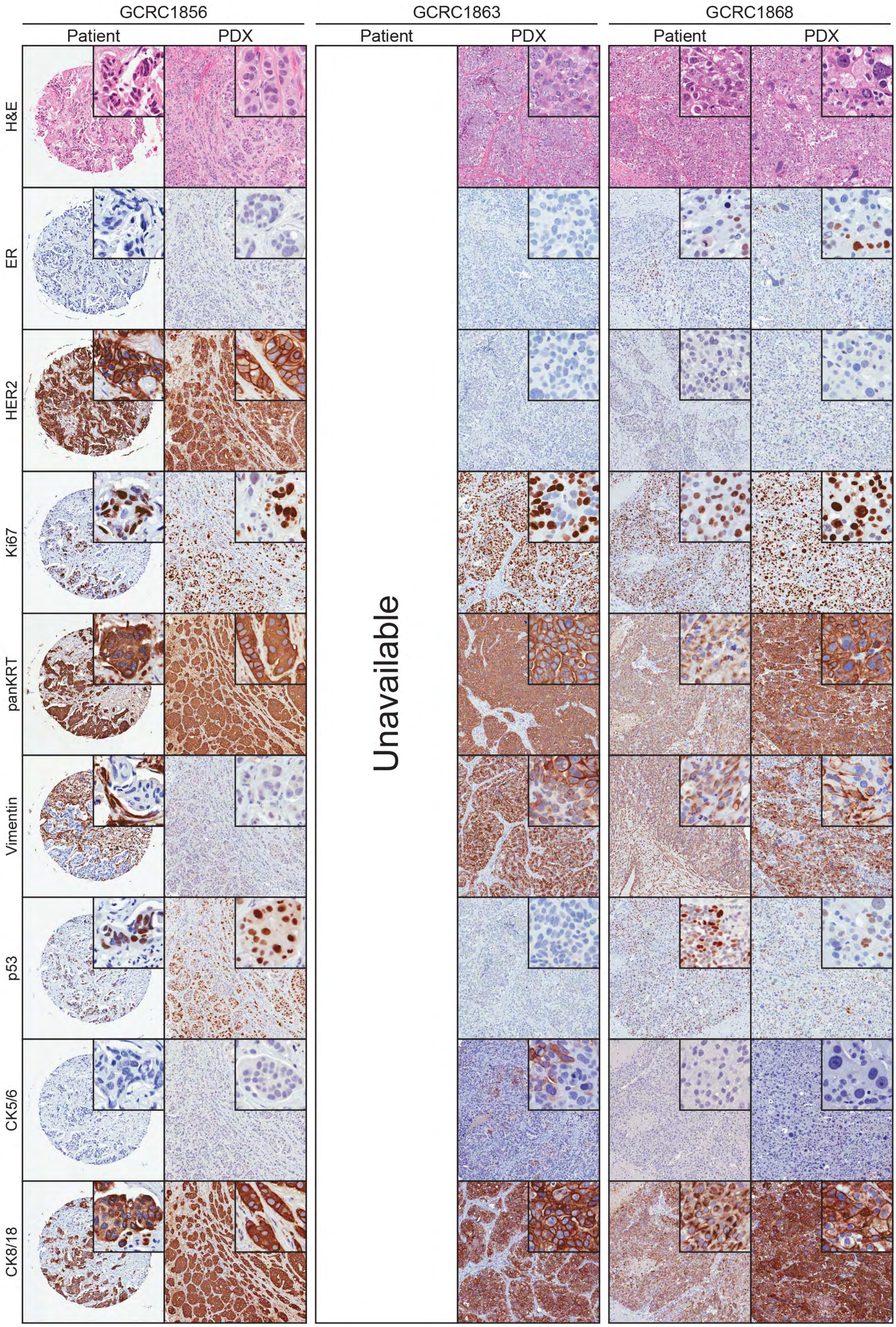

Supplementary Figure 3 (cont)

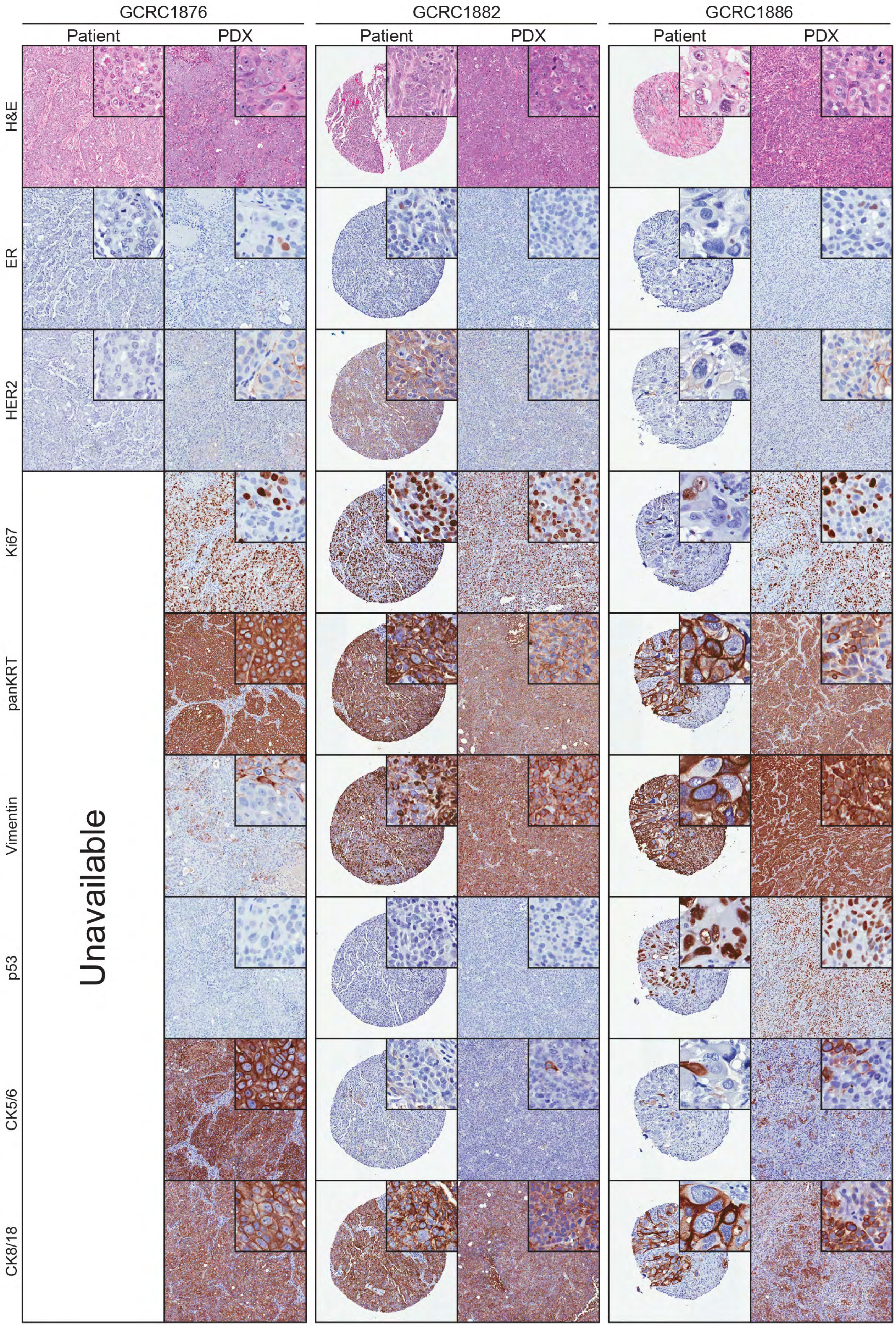

Supplementary Figure 3 (cont)

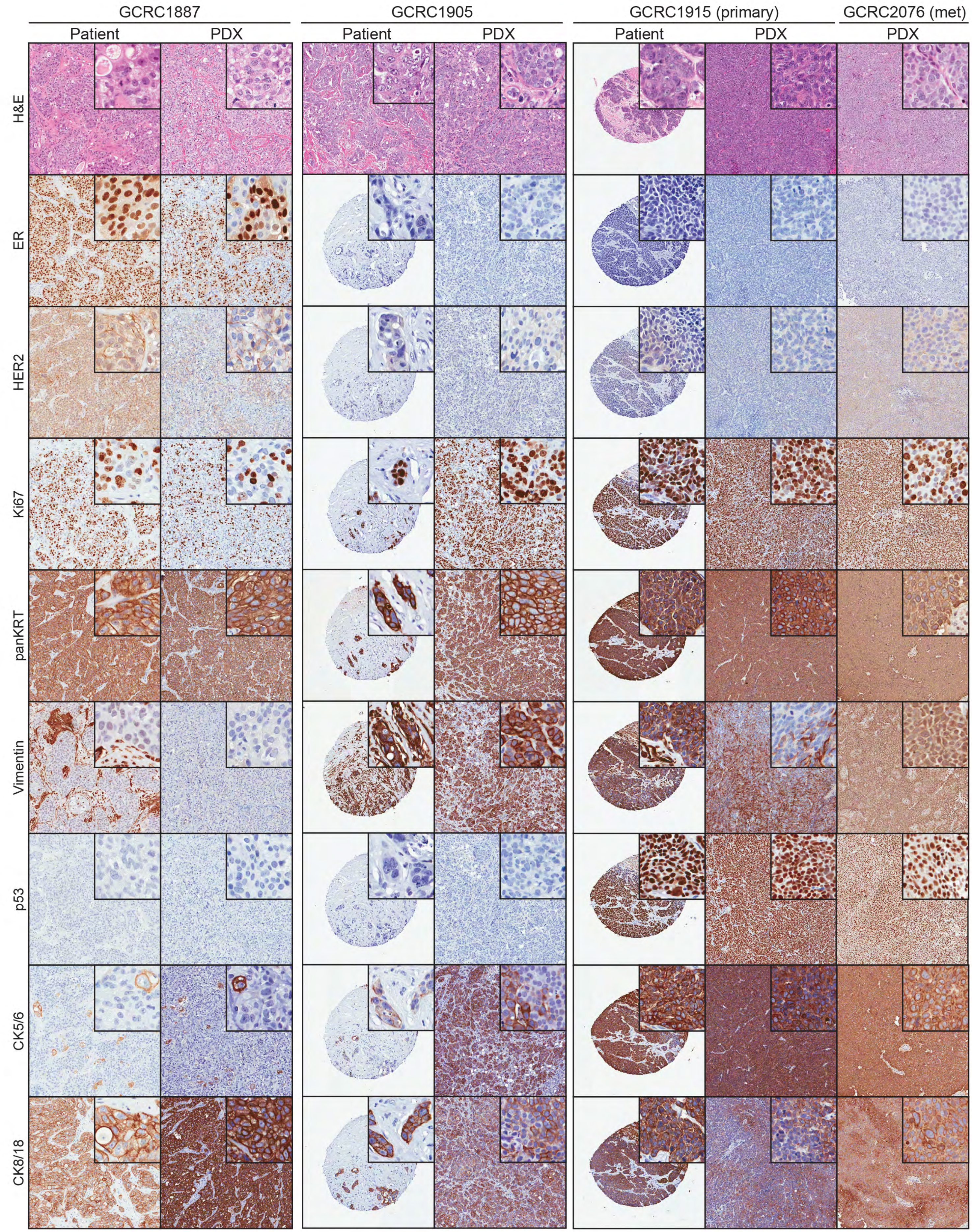

Supplementary Figure 3 (cont)

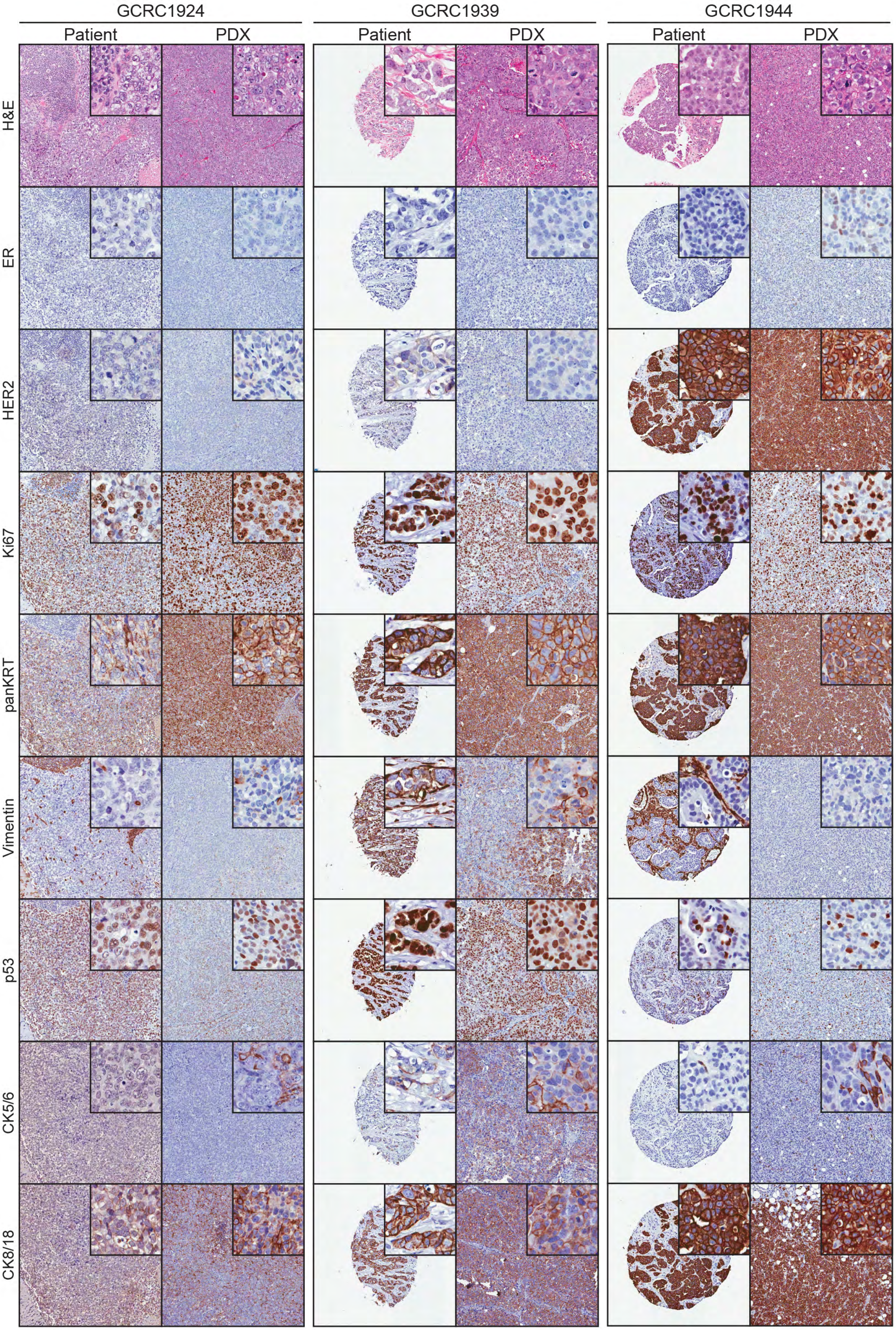

Supplementary Figure 3 (cont)

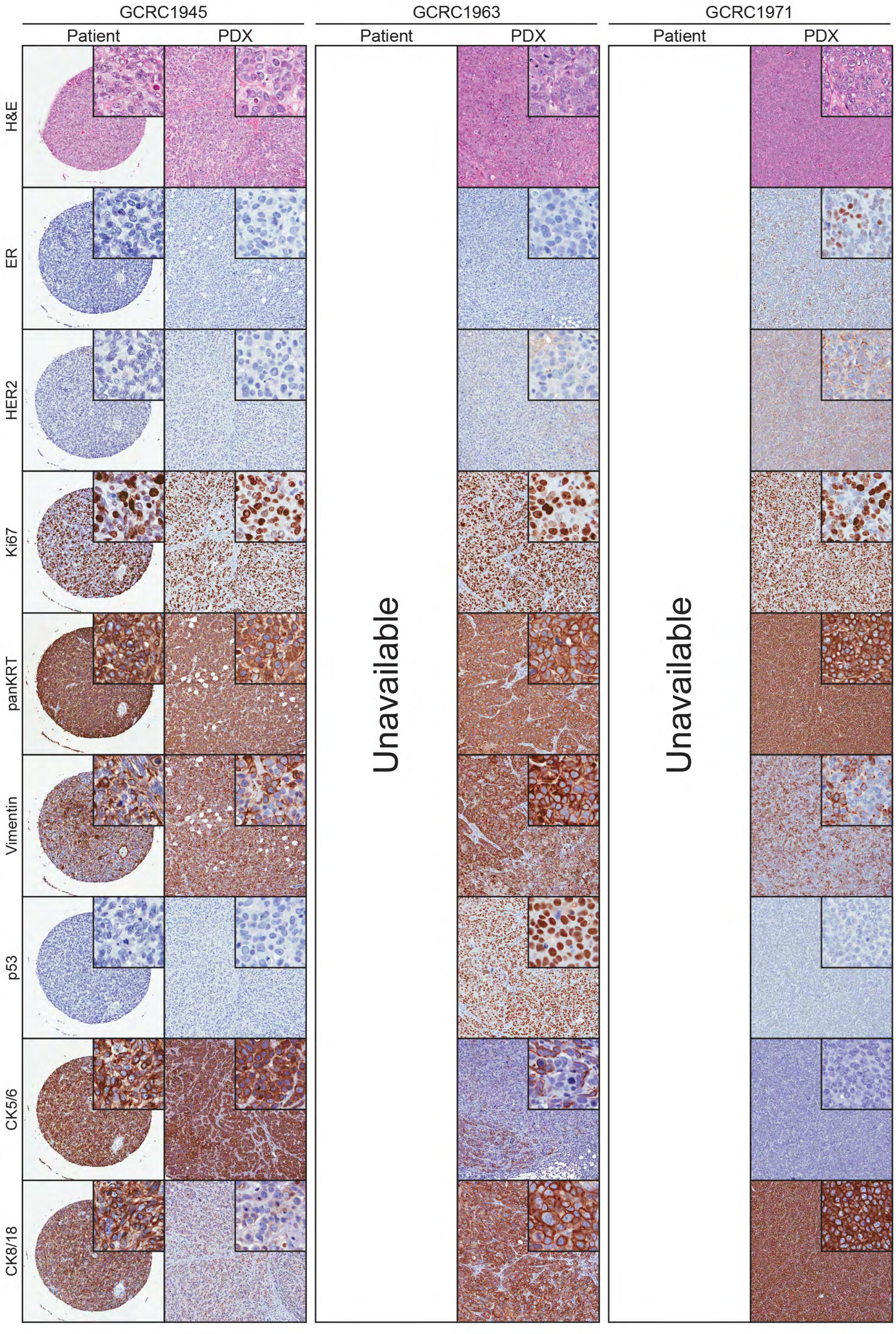

Supplementary Figure 3 (cont)

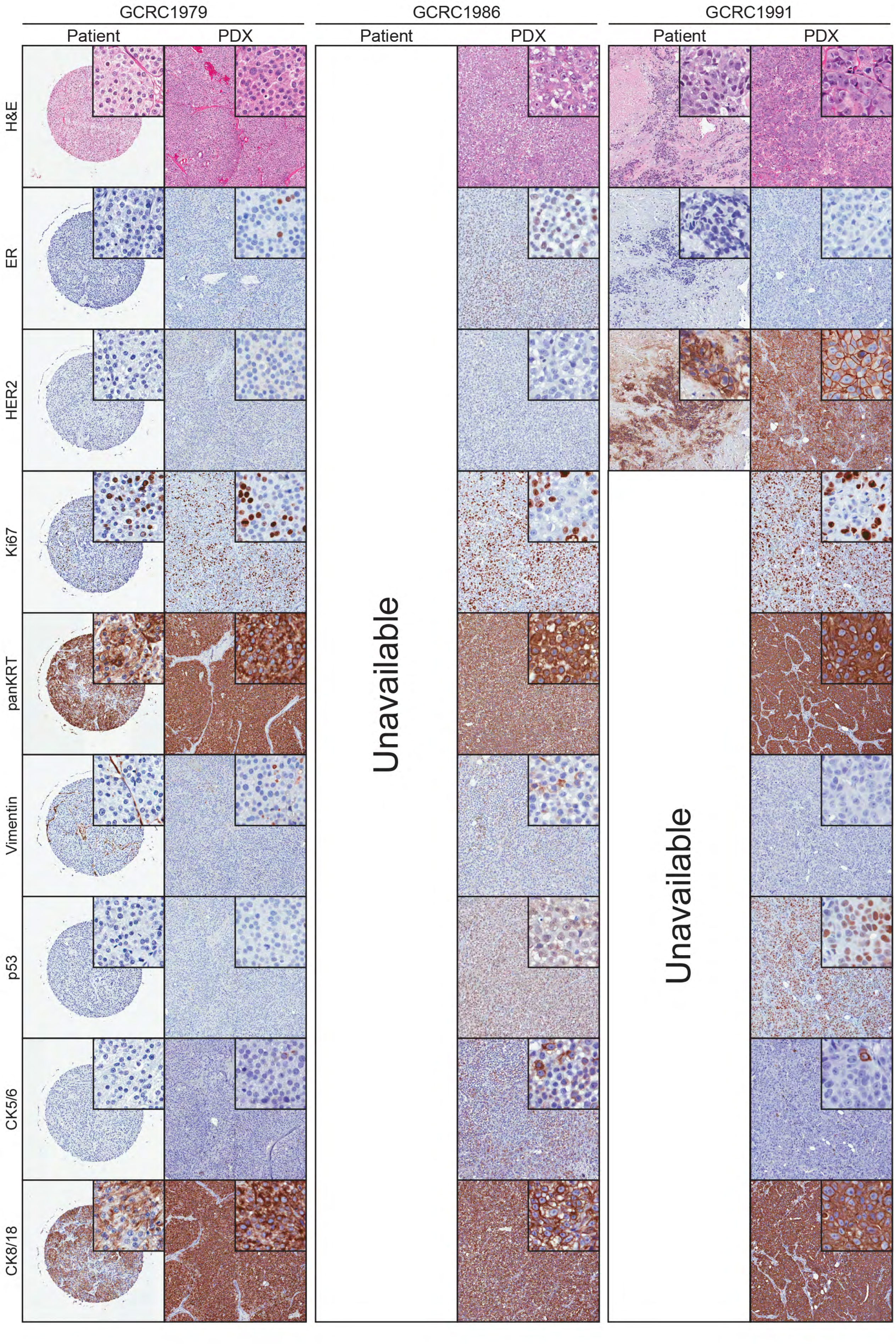

Supplementary Figure 3 (cont)

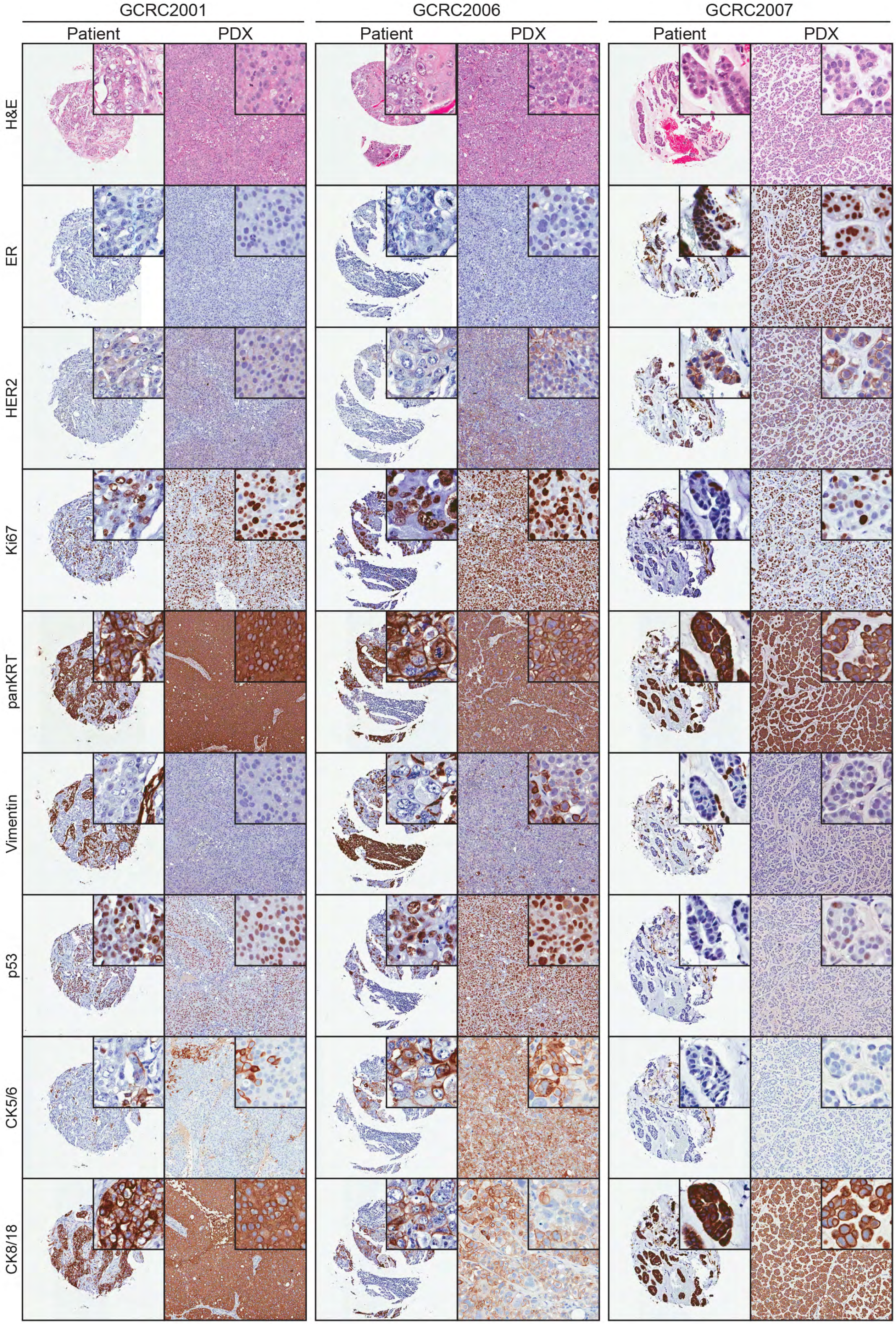

Supplementary Figure 3 (cont)

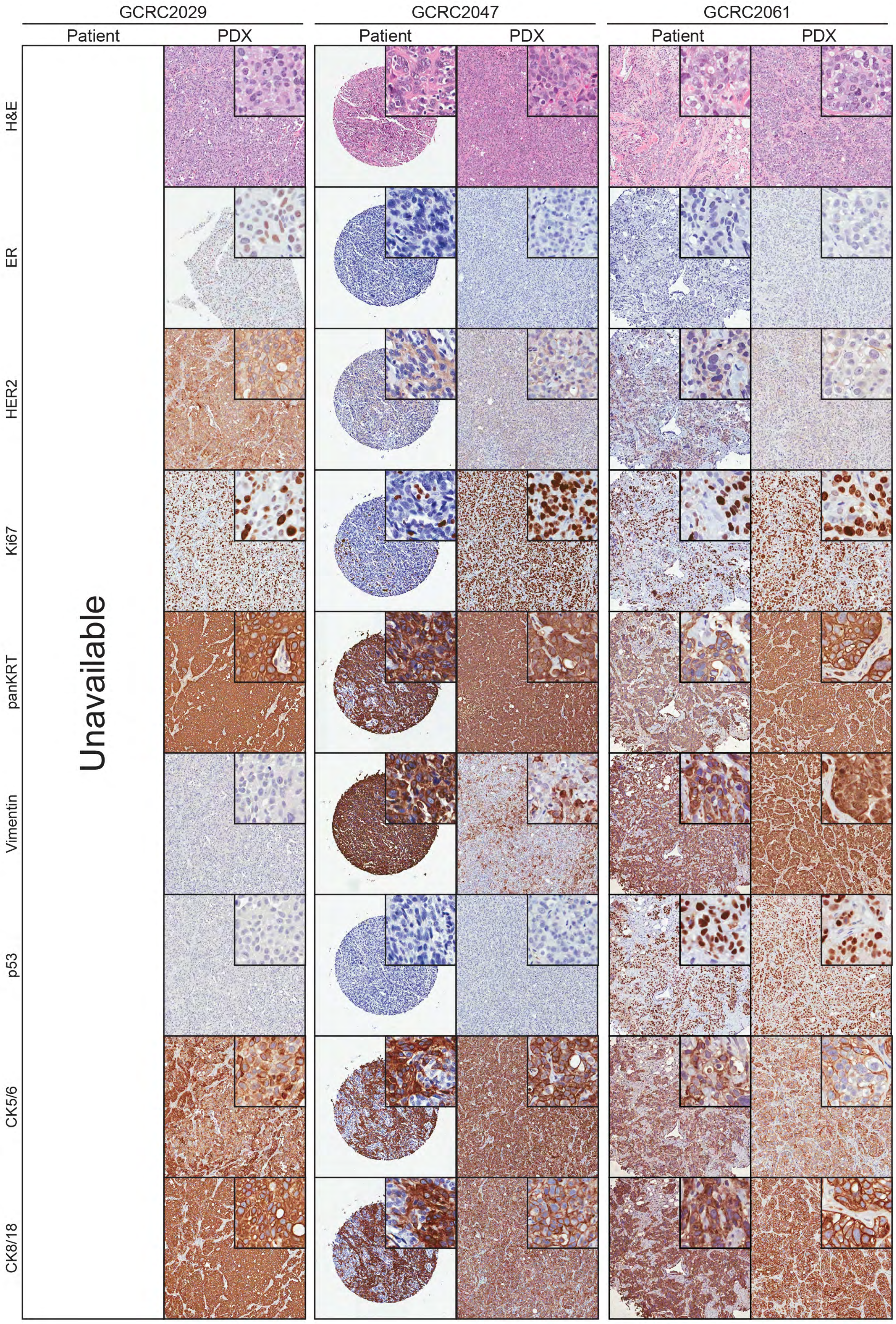

Supplementary Figure 3 (cont)

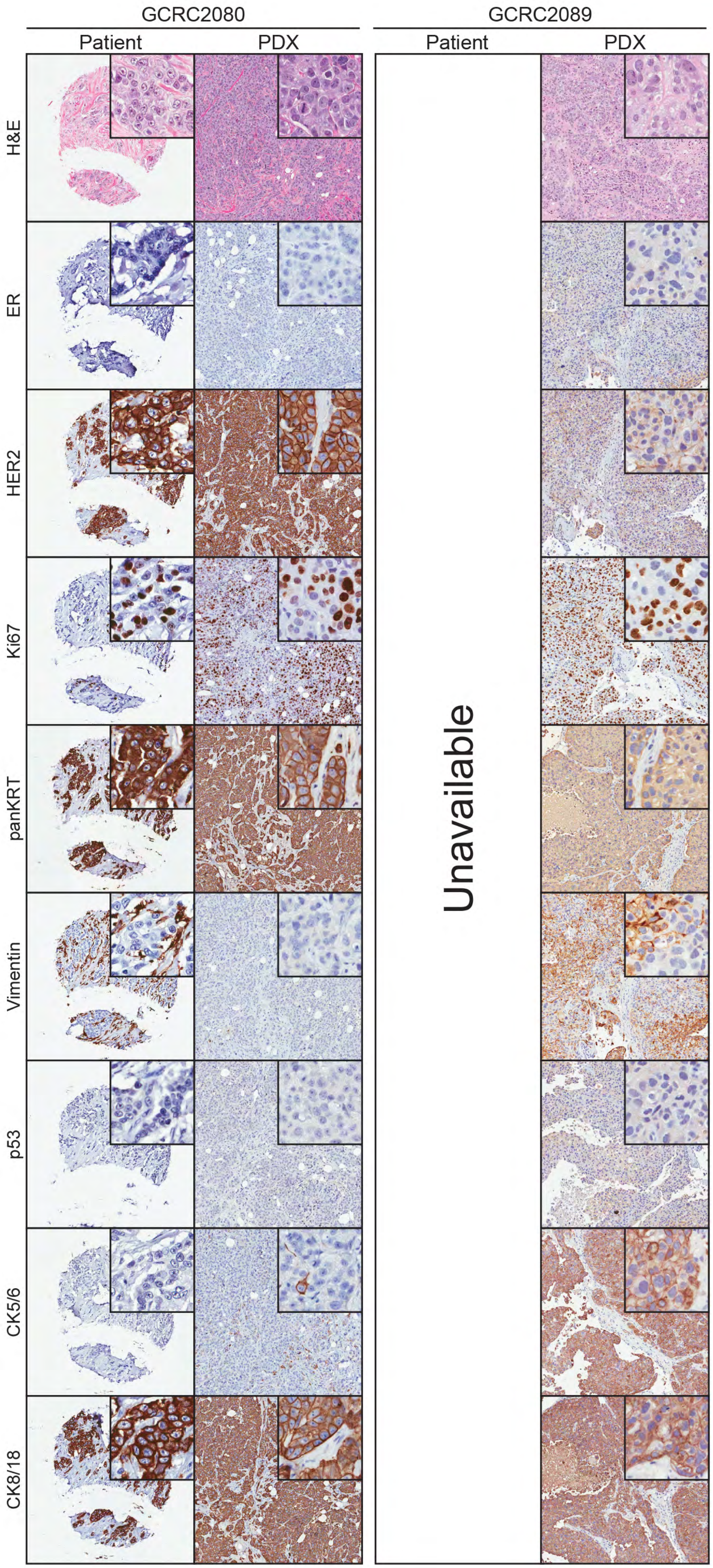

Supplementary Figure 3. Immunohistochemical staining of human tumors and PDXs. **A.** Representative immunohistochemical images of PDXs stained for H&E, estrogen receptor (ER), HER2, Ki67, pan-cytokeratin (pan-KRT), vimentin, p53, cytokeratin 5/6 (CK5/6) and cytokeratin 8/18 (CK8/18). Scale bar 200 μm, inset scale bar 50 μm.

Supplementary Figure 4

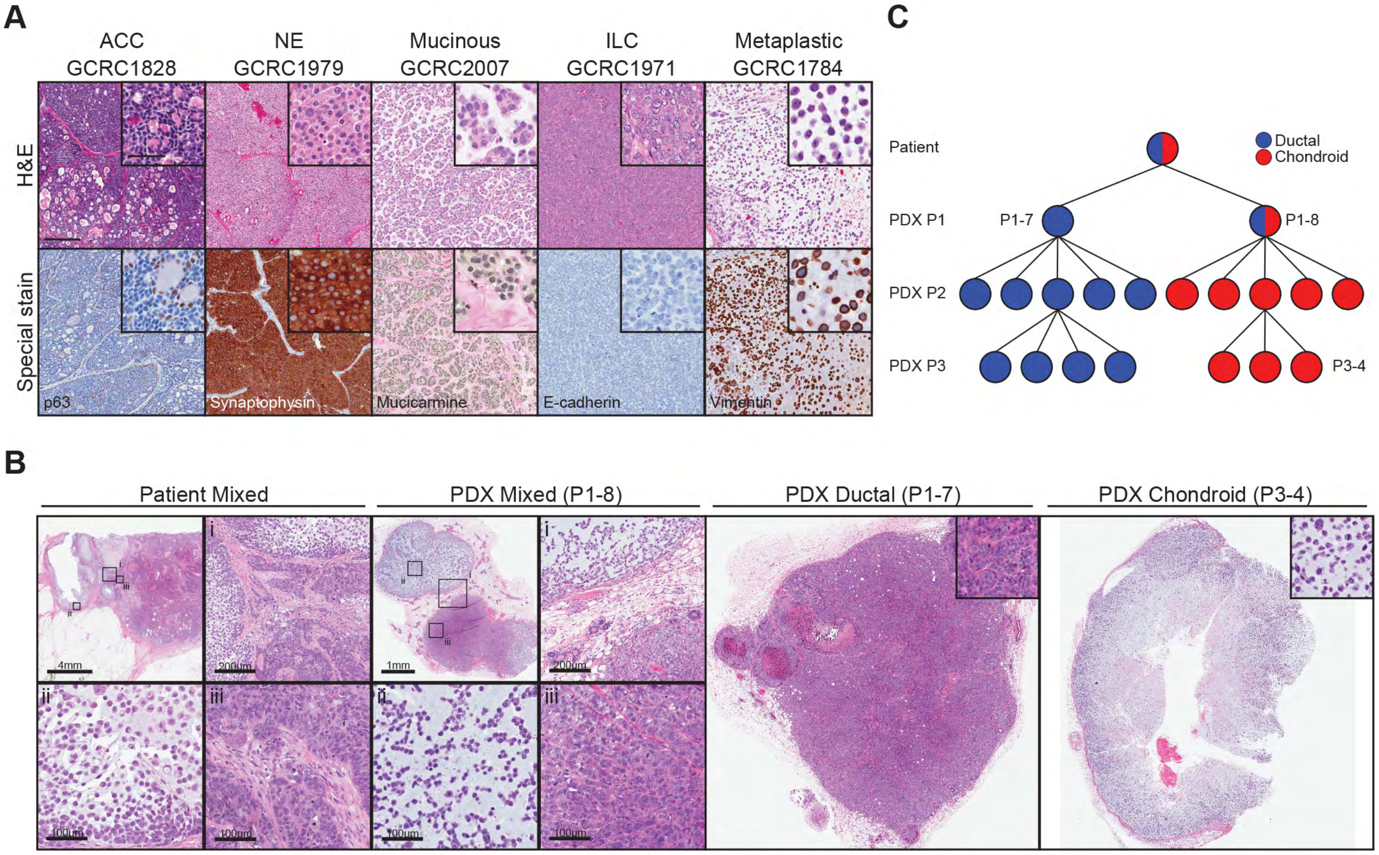

Supplementary Figure 4. Pathological features of rare histological variants. **A.** Representative immunohistochemical images of PDXs stained for special markers. The adenoid cystic carcinoma (ACC) stained for p63, neuroendocrine (NE) tumor for synaptophysin, mucinous tumor for mucicarmine, invasive lobular carcinoma for E-cadherin and metaplastic for vimentin. Scale bar 200 μm, inset scale bar 50 μm. **B.** H&E images from select regions of GCRC1784 patient and PDXs. The patient and PDX P1-8 tumors displayed mixed morphology with both ductal and chondroid components, while the P1-7 and P3-4 PDXs displayed pure ductal or chondroid histology, respectively. **C.** Graphical depiction of histology from GCRC1784 patient and PDX tumors over serial passaging.

Supplementary Figure 5

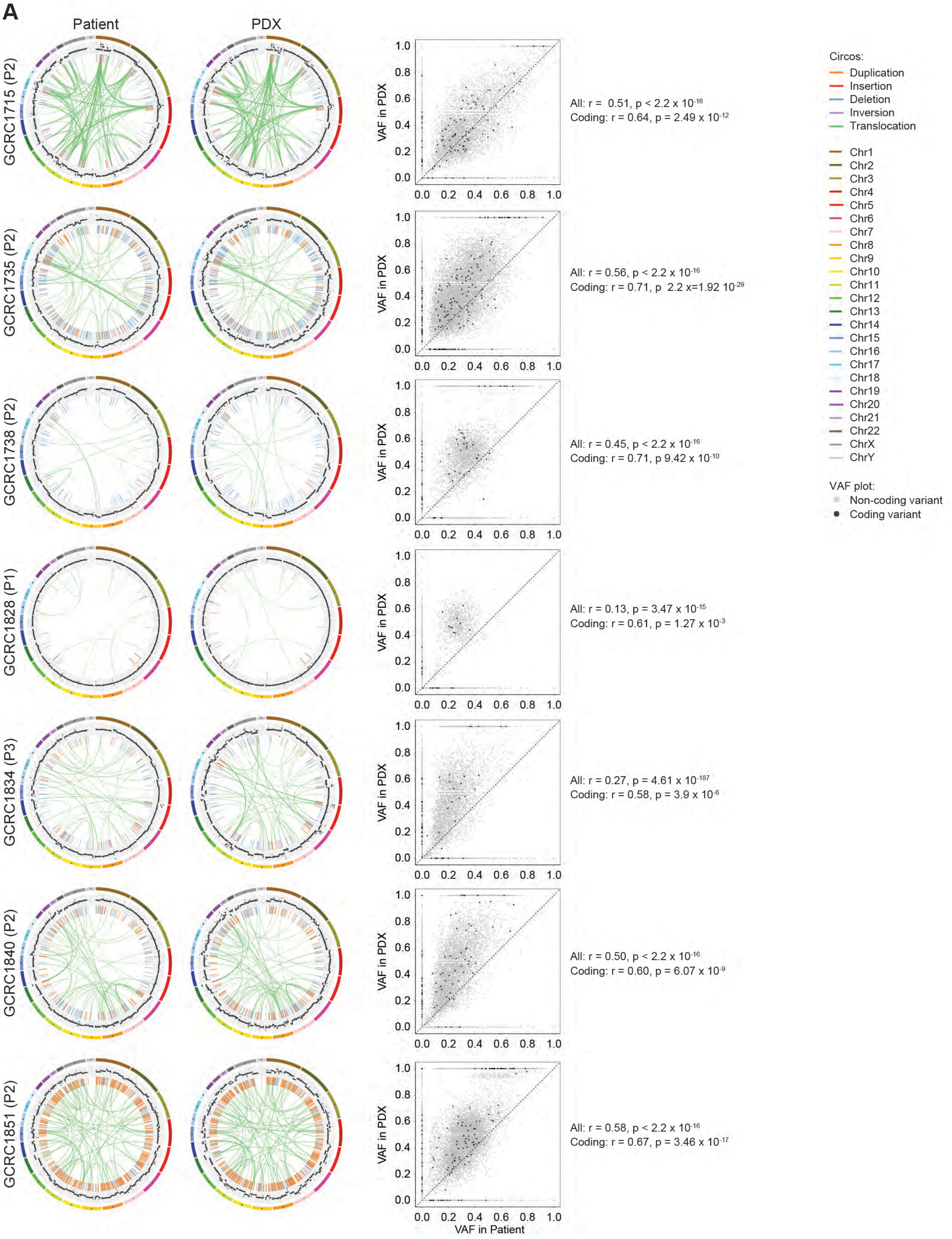

Supplementary Figure 5 (cont)

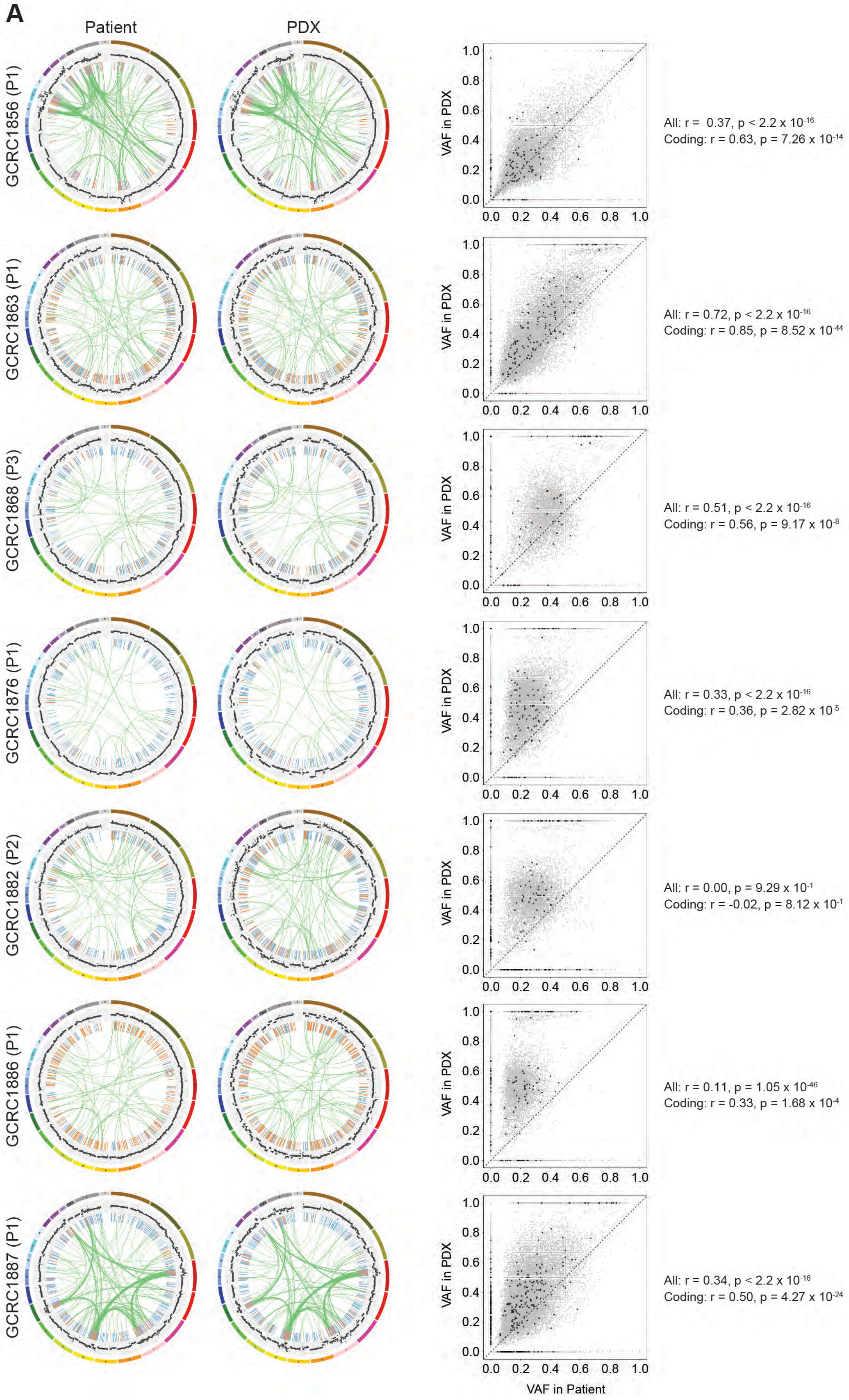

Supplementary Figure 5 (cont)

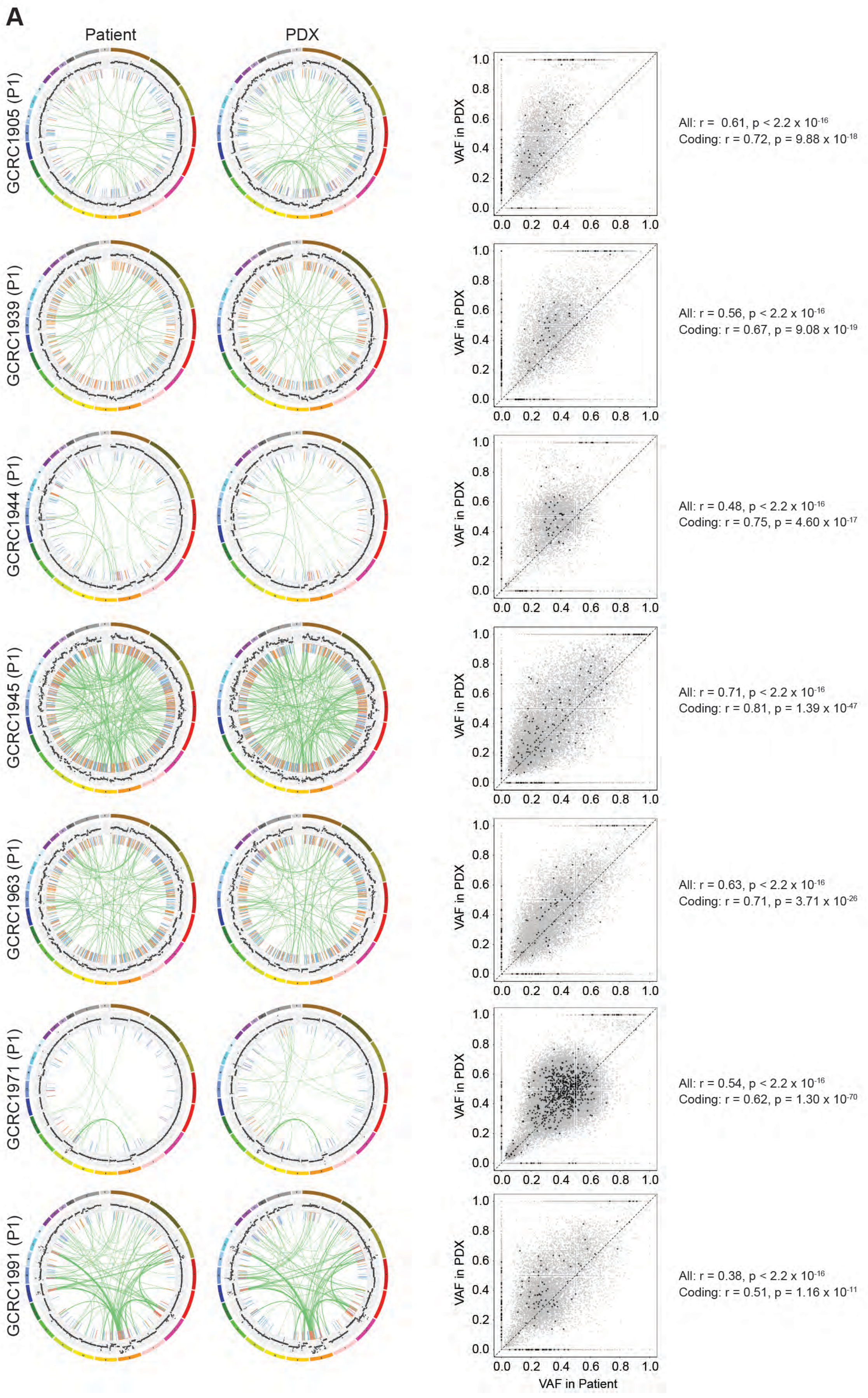

Supplementary Figure 5 (cont)

A

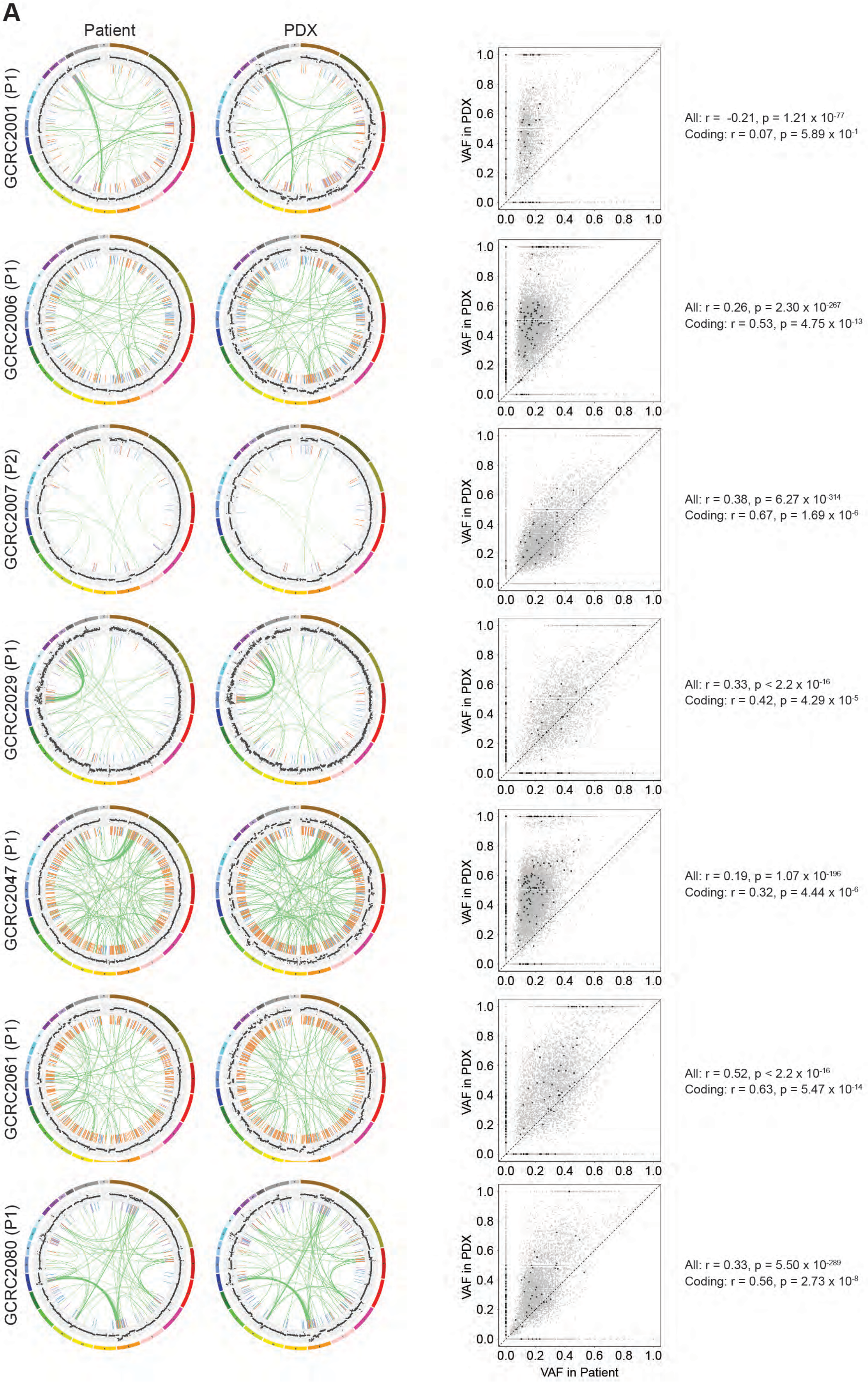

Supplementary Figure 5 (cont)

A

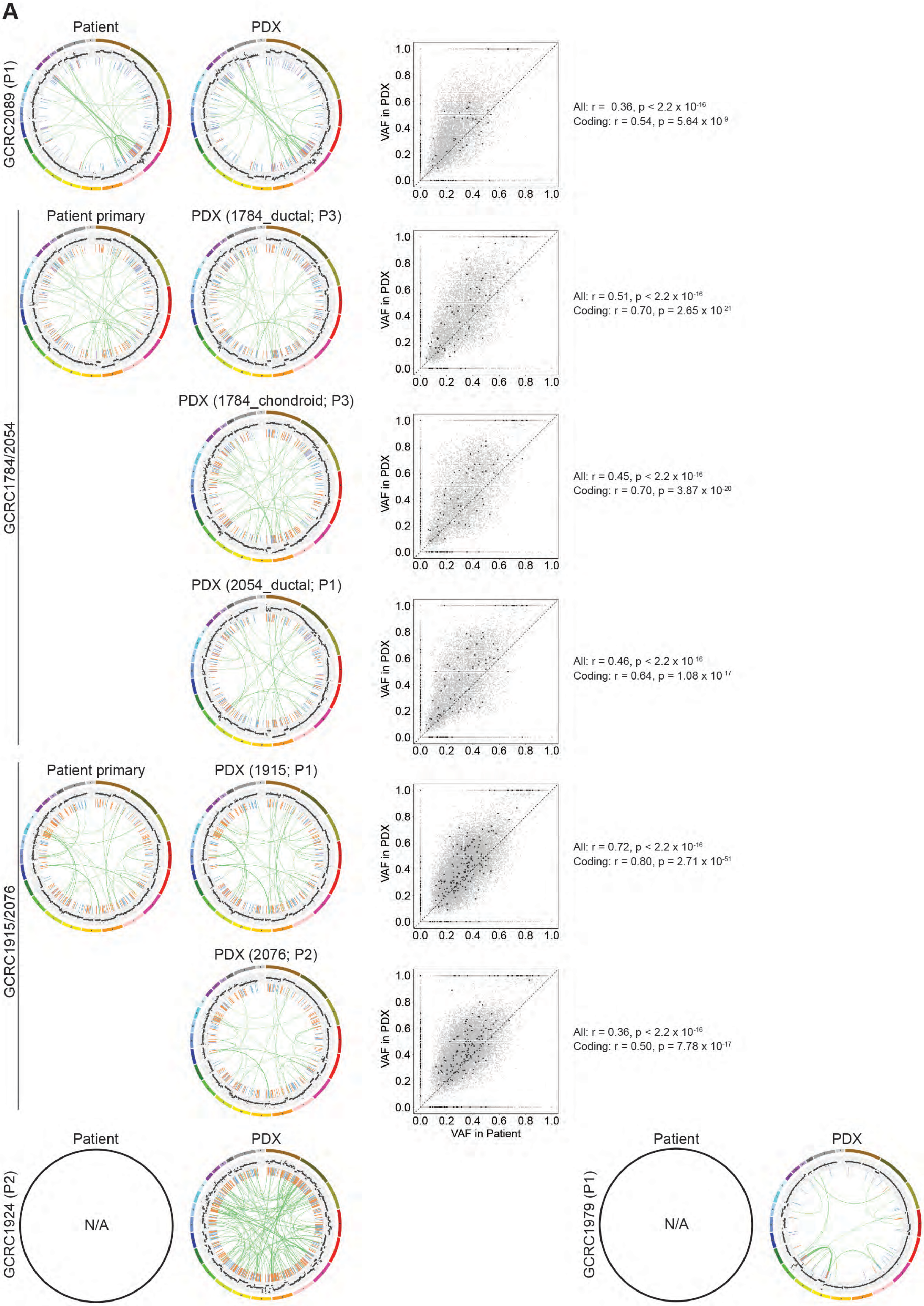

Supplementary Figure 5. PDX and patient tumor Circos plots and VAF scatter plots. **A.** Circos plots of patient and PDX tumors depicting structural variants (central part and inner ring) and copy number profile (outer ring) derived from whole-genome sequencing data. Variant allele frequency (VAF) scatter plots depicted on right with PDX on y-axis and patient tumor on x-axis. Passage generation (P#) for the PDX that underwent whole genome sequencing is denoted next to the model ID on the left.

Supplementary Figure 6

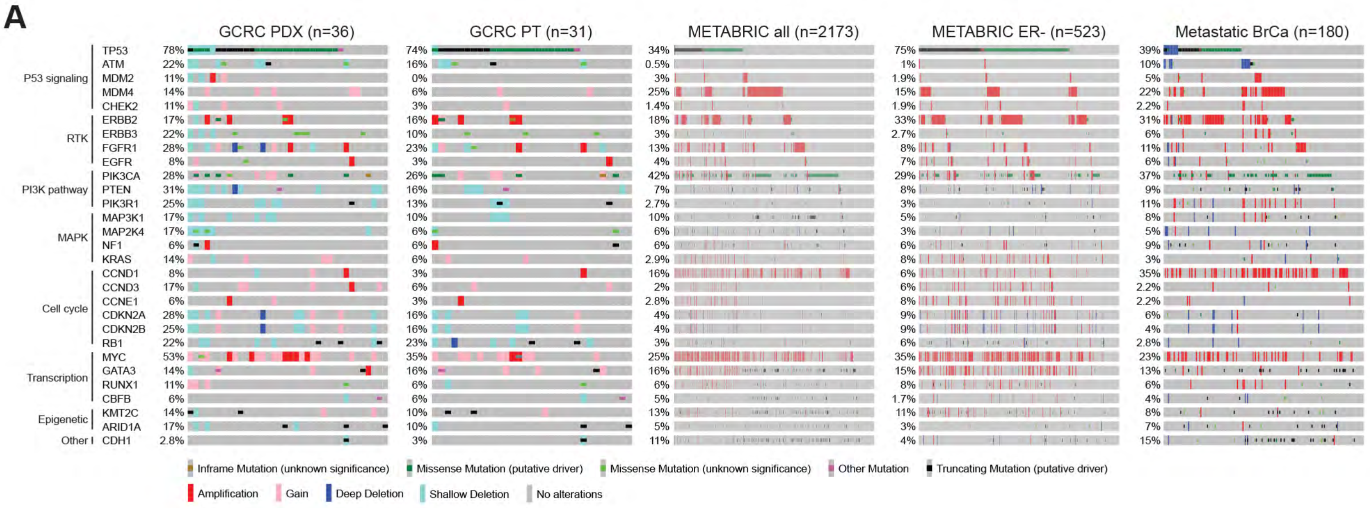

Supplementary Figure 6. Oncoprints for PDX, patients and public tumor datasets. **A.** Oncoprints of genes showing recurrent alterations in breast cancer across multiple datasets. Data from METABRIC and Metastatic Breast Cancer Project (provisional October 2018) retrieved from cBioPortal. PT, patient.

Supplementary Figure 7

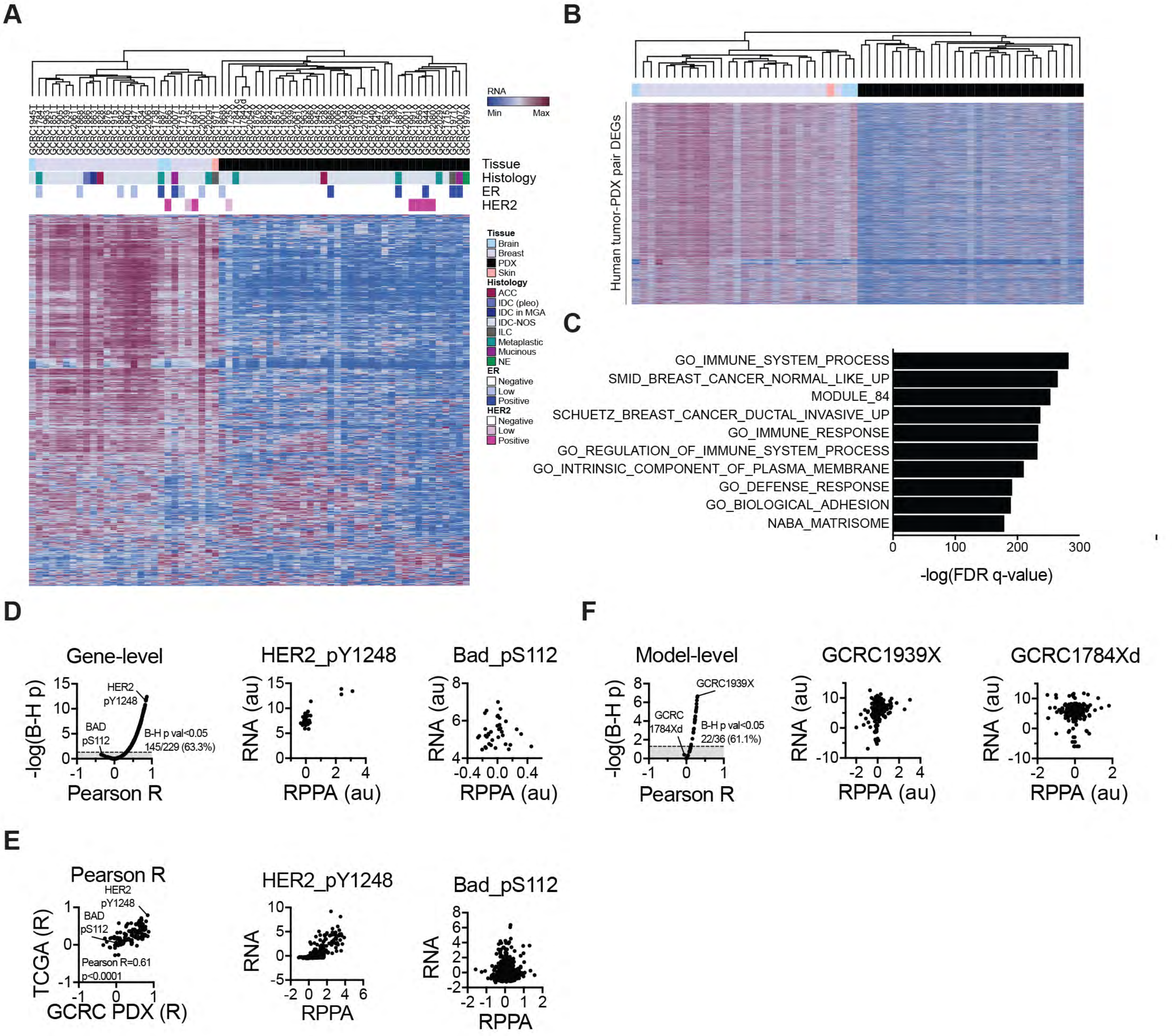

Supplementary Figure 7. RNA-sequencing and RPPA profiling of PDXs. **A.** Heatmap of unbiased hierarchical clustering across 66 samples (37 PDX, 29 patient tumors) prior to filtering of stromal genes. Clustering based on top 1000 most variable genes (inter-quartile range), with tissue origin, histopathological (histology, ER, HER2) and subtype (AIMS, absolute intrinsic molecular subtype) annotation (top). **B.** Heatmap of 3,037 differentially expressed (log2-fold higher in human tumors) genes between human tumor-PDX pairs (n=29 pairs). These were defined as stromal genes and were filtered for epithelial-centric analyses. **C.** Barchart of false discovery rate q-values for pathway analysis (mSigDB) on human tumor-PDX differentially expressed genes. **D.** Significance plot of correlations for genes/probes measured by RNA-sequencing and reverse-phase protein array (RPPA)(n=229). 145 of the 229 genes/probes showed significant correlations. B-H, Benjamini-Hochberg corrected p-values. On right, representative correlation plots for genes/probes which were highly significant (HER2_pY1248) and insignificant (Bad_pS112) across the PDX library (n=36). **E.** Scatter plot of RPPA/RNA-sequencing Pearson R values in the GCRC PDX and TCGA datasets (n=108). On right, representative correlation plots for genes/probes which were highly significant (HER2_pY1248) and insignificant (Bad_pS112) across the TCGA dataset (n=887). **F.** Significance plot of correlations for all genes/probes within individual PDXs (n=36). 22 of 36 PDXs displayed significantly correlated expression of genes/probes. On right, representative correlation plots for PDXs with highly significant (GCRC1939X) and insignificant (GCRC1784Xd) correlations across genes/probes (n=229).

Supplementary Figure 8

A

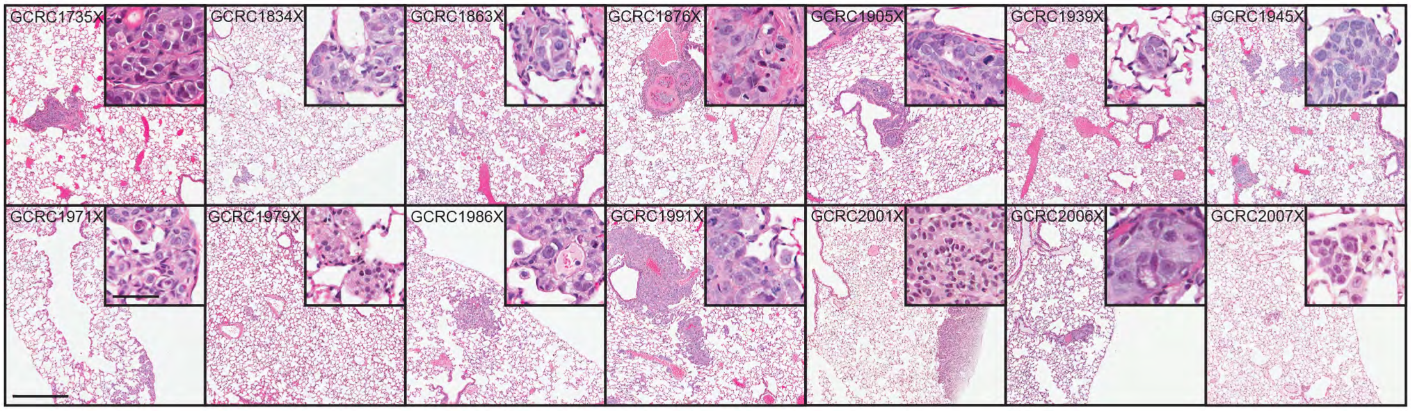

Supplementary Figure 8. PDX lung micrometastases. **A.** Representative H&E images of PDX lung micrometastases. Scale bar 400 µm, inset scale bar 50 µm.

Supplementary Figure 9

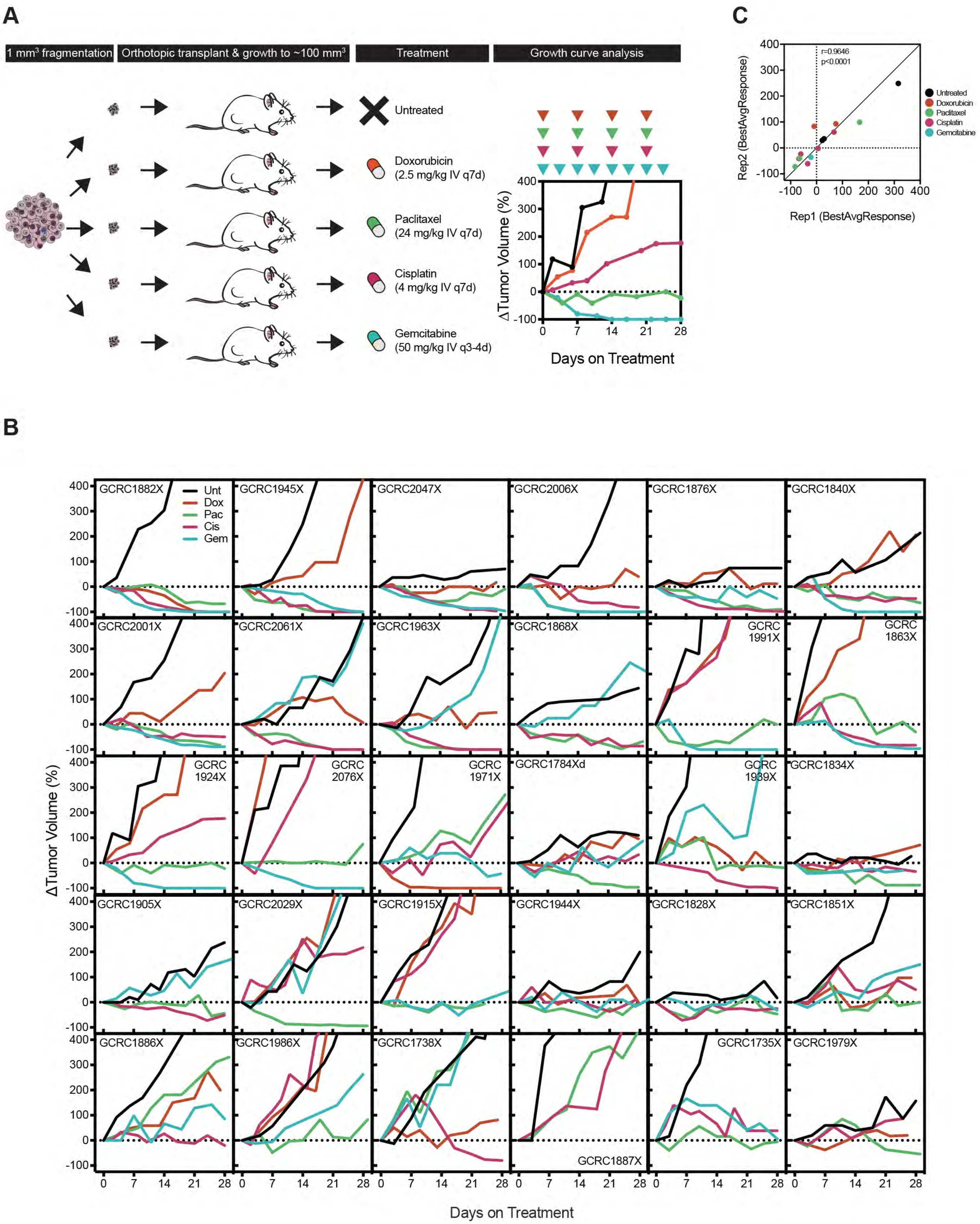

Supplementary Figure 9. PDX clinical trial for chemosensitivity profiling. **A.** Schematic of protocol for chemosensitivity studies using a 1x1x1 approach. **B.** Tumor growth curves for 1x1x1 chemosensitivity screen. Treatment arms include untreated, doxorubicin, paclitaxel, cisplatin or gemcitabine (n=1 per curve). **C.** Correlation plot of BestAvgResponse from replicates in PDX chemosensitivity clinical trial (n=13 replicates). Line of identity is represented.

Supplemental Tables

Supplementary Table 1. Chemosensitivity BestAvgResponse data.

PDX Model	Untreated	Doxorubicin	Gemcitabine	Cisplatin
GCRC1735X	326.6	NA	85.4	67.5
GCRC1738X	135.4	15.8	103.5	15.8
GCRC1784Xd	46.4	4.4	-5.8	3.5
GCRC1828X	18.6	NA	-5.3	-32.3
GCRC1834X	15.0	-7.4	-28.2	-15.4
GCRC1840X	51.7	24.6	-65.7	-33.4
GCRC1851X	106.0	8.3	10.8	49.1
GCRC1863X	557.8	184.4	-52.1	-28.3
GCRC1868X	94.2	NA	23.4	-54.4
GCRC1876X	12.8	24.8	-35.4	-67.7
GCRC1882X	220.5	-49.3	-74.5	-62.0
GCRC1886X	133.3	36.5	31.5	6.1
GCRC1887X	352.1	NA	NA	72.7
GCRC1905X	38.6	NA	28.9	-29.1
GCRC1915X	178.3	93.4	-15.6	124.2
GCRC1924X	235.5	123.8	-76.4	36.4
GCRC1939X	460.5	39.5	122.2	-68.8
GCRC1944X	33.2	10.5	-1.4	2.4
GCRC1945X	85.0	12.9	-57.5	-60.5
GCRC1963X	76.0	18.4	7.4	-66.0
GCRC1971X	316.3	-83.4	8.5	-0.1
GCRC1979X	35.7	-6.1	NA	12.6
GCRC1986X	96.1	91.6	34.1	113.2
GCRC1991X	531.3	160.7	-73.6	152.8
GCRC2001X	135.3	18.9	-53.8	-28.1
GCRC2006X	49.6	-8.6	-72.3	-30.6
GCRC2029X	56.6	95.8	54.0	87.7
GCRC2047X	29.6	-12.3	-62.0	-58.8
GCRC2061X	57.1	50.8	74.6	-72.0
GCRC2076X	240.4	619.8	-75.5	92.4

Supplementary Table 2. Actionable gene list.

**WGS (OncoKB, ESMO/ESCAT)**	**RNA (DEPO)**	**RPPA (Akbani et al. 2014 Nat Comm, DEPO)**
**OncoKB_Level 1**	AR (AR modulator/antagonist sensitivity)	ACC_pS79-R-V (ACC inhibitor sensitivity)
ERBB2 amplification (ERBB2 inhibitor sensitivity)	ATM (PARP inhibitor sensitivity)	ACC1-R-C (ACC inhibitor sensitivity)
NTRK fusion (Larotrectanib sensitivity)	AURKA (AURK inhibitor sensitivity)	Akt_pS473-R-V (Akt inhibitor sensitivity)
MSI-H (PD-1/PD-L1 blockade sensitivity)	CD274 (PD-1/PD-L1 blockade sensitivity)	Akt_pT308-R-V (Akt inhibitor sensitivity)
**OncoKB_Level 2A**	CD30 (brentuximab sensitivity)	Akt-R-V (Akt inhibitor sensitivity)
Germline BRCA1/2 mutation (PARP inhibitor sensitivity)	CRKL (EGFR inhibitor resistance)	AMPKa_pT172-R-C (metformin sensitivity)
**OncoKB_Level 3A**	DLL3 (DLL3 ADC sensitivity)	AMPKa-R-C (metformin sensitivity)
AKT1 mutation (AZD5363 sensitivity)	EGFR (EGFR inhibitor resistance)	AR-R-V (AR modulator/antagonist sensitivity)
ERBB2 mutation (Neratinib sensitivity)	EPHA2 (dasatanib sensitivity)	ARID1A-R-C (PI3K inhibitor sensitivity)
ESR1 mutation (AZD9496, fulvestrant sensitivity)	EPHA3 (EPHA3 inhibitor sensitivity)	ATM_pS1981-R-V (ATM inhibitor sensitivity)
NTRK fusion (Entrectinib sensitivity)	ERBB2 (ERBB2 inhibitor sensitivity)	ATM-R-V (ATM inhibitor sensitivity)
PIK3CA mutation (PI3K inhibitor sensitivity)	ERCC1 (cisplatin sensitivity)	ATR_pS428-R-C (ATR inhibitor sensitivity)
**OncoKB_Level 4**	ESR1 (SERM/SERD/AI sensitivity)	B-Raf_pS445-R-V (BRAF inhibitor sensitivity)
ATM mutation (PARP inhibitor sensitivity)	FBXW7 (anti-tubulin resistance)	B-Raf-R-C (BRAF inhibitor sensitivity)
BRAF mutation (PLX8394 sensitivity)	IGF1R (erlotinib resistance)	BRD4-R-V (JQ1 sensitivity)
CDK12 truncating mutation (PD-1/PD-L1 blockade sensitivity)	IL2RA (denileukin diftitox sensitivity)	c-Kit-R-V (imatinib sensitivity)
CDKN2A mutation (CDK4/6 inhibitor sensitivity)	KIT (imatinib sensitivity)	c-Met_pY1234_Y1235-R-V (MET inhibitor sensitivity)
FGFR1/2/3 mutation (FGFR inhibitor sensitivity)	LRP1B (liposomal doxorubicin sensitivity)	c-Myc-R-C (PIM1K/FAO inhibitor sensitivity)
KRAS mutation (MEK inhibitor sensitivity)	MCL1 (anti-tubulin resistance)	Chk1_pS296-R-V (CHK1 inhibitor sensitivity)
MET fusion (crizotinib sensitivity)	MS4A1 (anti-CD20 sensitivity)	Chk2_pT68-R-C (CHK2 inhibitor sensitivity)
MTOR mutation (MTOR inhibitor sensitivity)	NF2 (FAK inhibitor sensitivity)	EGFR_pY1173-R-V (EGFR inhibitor sensitivity)
NF1 mutation (MEK inhibitor sensitivity)	PAK1 (PAK1 inhibitor sensitivity)	EGFR-R-V (EGFR inhibitor sensitivity)
PTEN deletion/mutation (PI3K inhibitor sensitivity)	PDGFRA (PDGFR inhibitor resistance)	ER-a_pS118-R-V (SERM/SERD/AI sensitivity)
SMARCB1 mutation (EZH2 inhibitor sensitivity)	PDPK1 (PI3K inhibitor sensitivity)	ER-R-V (SERM/SERD/AI sensitivity)
**ESMO/ESCAT_Level 1A**	PGR (SERM/SERD/AI sensitivity)	FAK_pY397-R-V (FAK inhibitor sensitivity)
ERBB2 amplification (ERBB2 inhibitor sensitivity)	PRKCH (trametinib sensitivity)	FAK-R-C (FAK inhibitor sensitivity)
Germline BRCA1/2 mutation (PARP inhibitor sensitivity)	PTEN (anti-EGFR mAbs resistance)	FASN-R-V (FAS inhibitor sensitivity)
PIK3CA mutation (PI3K inhibitor sensitivity)	SYK (SYK inhibitor sensitivity)	HER2_pY1248-R-C (ERBB2 inhibitor sensitivity)
**ESMO/ESCAT_Level 1C**		HER3_pY1289-R-C (ERBB3 mAb sensitivity)
MSI-H (PD-1/PD-L1 blockade sensitivity)		HER3-R-V (ERBB3 mAb sensitivity)
NTRK fusion (Larotrectanib sensitivity)		IGF1R_pY1135_Y1136-R-V (IGFR inhibitor sensitivity)
**ESMO/ESCAT_Level 2A**		IGFRb-R-C (IGFR inhibitor sensitivity)
ESR1 mutation (AZD9496, fulvestrant sensitivity)		Mcl-1-R-V (anti-tubulin resistance)
PTEN deletion/mutation (PI3K inhibitor sensitivity)		MEK1_pS217_S221-R-V (MEK inhibitor sensitivity)
AKT1 mutation (AZD5363 sensitivity)		MEK1-R-V (MEK inhibitor sensitivity)
ERBB2 mutation (Neratinib sensitivity)		Merlin-R-C (FAK inhibitor sensitivity)
**ESMO/ESCAT_Level 3A**		mTOR_pS2448-R-C (mTOR inhibitor sensitivity)
MDM2 amplification (MDM2 inhibitor)		mTOR-R-V (mTOR inhibitor sensitivity)
Somatic BRCA1/2 mutation (PARP inhibitor sensitivity)		Notch1-R-V (Gamma-secretase/Notch inhibitor sensitivity)
**ESMO/ESCAT_Level 3B**		PAK1-R-V (PAK1 inhibitor sensitivity)
ERBB3 mutation (Neratinib sensitivity)		PAR-R-C (PARP inhibitor sensitivity)
**ESMO/ESCAT_Level 4A**		PARP-R-V (PARP inhibitor sensitivity)
ARID1A/B mutation (EZH2 inhibitor sensitivity)		PD-L1-R-C (PD-1/PD-L1 blockade sensitivity)
ATR/ATM/PALB2 mutation (PARP/ATR inhibitor sensitivity)		PDGFR-b-R-V (PDGFR inhibitor sensitivity)
CDH1 mutation (ROS1 inhibitor sensitivity)		PI3K-p110-a-R-C (PI3K inhibitor sensitivity)
INPP4B loss (PI3K inhibitor sensitivity, with PTEN loss)		PI3K-p85-R-V (PI3K inhibitor sensitivity)
MAP2K4/MAP3K1 mutation (MEK inhibitor sensitivity)		PR-R-V (SERM/SERD/AI sensitivity)
MYC amplification (PIM1 inhibitor sensitivity)		PTEN-R-V (PI3K inhibitor sensitivity)
NF1 mutation (MEK inhibitor sensitivity)		Src_pY416-R-V (SRC inhibitor sensitivity)
PIK3R1 mutation (MEK inhibitor sensitivity)		Src_pY527-R-V (SRC inhibitor sensitivity)
SF3B1 mutation (H3B-8800 sensitivity)		Stat3_pY705-R-V (STAT inhibitor sensitivity)
TP53 mutation (APR-246 sensitivity)		Stat3-R-C (STAT inhibitor sensitivity)
		XRCC1-R-C (ATM inhibitor sensitivity)
