# Supplementary material for: Chemogenomic profiling of breast cancer patient-derived xenografts reveals targetable vulnerabilities for difficult-to-treat tumors
Source: Commun Biol. 2020 Jun 16;3:310. doi: 10.1038/s42003-020-1042-x (PMC7298048; doi:10.1038/s42003-020-1042-x)
Supplement: Supplementary file 3 — Reporting Summary [file 42003_2020_1042_MOESM3_ESM.pdf]

nature research

Corresponding author(s): Morag Park

Last updated by author(s): May 1, 2020

Reporting Summary

Nature Research wishes to improve the reproducibility of the work that we publish. This form provides structure for consistency and transparency in reporting. For further information on Nature Research policies, see Authors & Referees and the Editorial Policy Checklist.

Statistics

For all statistical analyses, confirm that the following items are present in the figure legend, table legend, main text, or Methods section.

n/a	Confirmed	
☐	☒	The exact sample size (*n*) for each experimental group/condition, given as a discrete number and unit of measurement
☐	☒	A statement on whether measurements were taken from distinct samples or whether the same sample was measured repeatedly
☐	☒	The statistical test(s) used AND whether they are one- or two-sided<br>*Only common tests should be described solely by name; describe more complex techniques in the Methods section.*
☒	☐	A description of all covariates tested
☒	☐	A description of any assumptions or corrections, such as tests of normality and adjustment for multiple comparisons
☐	☒	A full description of the statistical parameters including central tendency (e.g. means) or other basic estimates (e.g. regression coefficient) AND variation (e.g. standard deviation) or associated estimates of uncertainty (e.g. confidence intervals)
☐	☒	For null hypothesis testing, the test statistic (e.g. *F*, *t*, *r*) with confidence intervals, effect sizes, degrees of freedom and *P* value noted<br>*Give P values as exact values whenever suitable.*
☒	☐	For Bayesian analysis, information on the choice of priors and Markov chain Monte Carlo settings
☐	☒	For hierarchical and complex designs, identification of the appropriate level for tests and full reporting of outcomes
☐	☒	Estimates of effect sizes (e.g. Cohen's *d*, Pearson's *r*), indicating how they were calculated

Our web collection on statistics for biologists contains articles on many of the points above.

Software and code

Policy information about availability of computer code

Data collection

No software was used for data collection.

Data analysis

Whole genome sequencing analysis:
Trimming: Trimmomatic (version 0.35)
Alignment: BWA-MEM (version 0.7.15)
Alignment recalibration: GATK (version 3.8)
Duplicates marking: Picard (version 2.9).
Filtering mouse reads: Disambiguate (version 1.0)
Somatic mutation calling: GATK's Mutect2 algorithm
Structural variant calling: Manta (version 1.5)
Copy number variation: SCoNEs (version 2.1)
Variant annotation: SnpEff (version 4.3)
Cancer pathogenicity: CRAVAT (version 5.2.4)
Oncoprints: cBioPortal

RNA sequencing analysis:
Trimming: Trimmomatic (version 0.35)
Alignment: STAR
Filtering mouse reads: Disambiguate (version 1.0)
Read counts: HTSeq
Normalization: edgeR (version 3.22.5)
Conversion to log counts per million: limma (version 3.36.5)
Pathway analysis: ssGSEA on GenePattern (version 3.9.10)

For manuscripts utilizing custom algorithms or software that are central to the research but not yet described in published literature, software must be made available to editors/reviewers. We strongly encourage code deposition in a community repository (e.g. GitHub). See the Nature Research guidelines for submitting code & software for further information.

Data

Policy information about availability of data

All manuscripts must include a data availability statement. This statement should provide the following information, where applicable:

- Accession codes, unique identifiers, or web links for publicly available datasets
- A list of figures that have associated raw data
- A description of any restrictions on data availability

Whole-genome sequencing data has been deposited in the Sequence Read Archive under the accession PRJNA594000. RNA sequencing data has been deposited in the Gene Expression Omnibus under the accession GSE142767.

Field-specific reporting

Please select the one below that is the best fit for your research. If you are not sure, read the appropriate sections before making your selection.

☒ Life sciences ☐ Behavioural & social sciences ☐ Ecological, evolutionary & environmental sciences

For a reference copy of the document with all sections, see nature.com/documents/nr-reporting-summary-flat.pdf

Life sciences study design

All studies must disclose on these points even when the disclosure is negative.

Sample size	This is a descriptive study on breast cancer patient-derived xenografts. Sample size was not included for number of patients included in study.
Data exclusions	No data was excluded from this study.
Replication	No sample replicates were performed for sequencing studies. In the 1x1x1 chemosensitivity study, the concordance between 13 replicates was high (r=0.9646, p<0.0001), as described in Fig. S9B.
Randomization	In the 1x1x1 chemosensitivity study, tumor-bearing mice were enrolled into treatment arms on when tumor volume reached 100 mm3. They were randomized to treatment arm using a randomization equation in Excel.
Blinding	Tumor measurements were conducted by two animal technicians who were not aware of the experimental design, treatment allocations and analytic methods.

Reporting for specific materials, systems and methods

We require information from authors about some types of materials, experimental systems and methods used in many studies. Here, indicate whether each material, system or method listed is relevant to your study. If you are not sure if a list item applies to your research, read the appropriate section before selecting a response.

Materials & experimental systems

n/a	Involved in the study	
☐	☒	Antibodies
☒	☐	Eukaryotic cell lines
☒	☐	Palaeontology
☐	☒	Animals and other organisms
☐	☒	Human research participants
☒	☐	Clinical data

Methods

n/a	Involved in the study	
☒	☐	ChIP-seq
☒	☐	Flow cytometry
☒	☐	MRI-based neuroimaging

Antibodies

Antibodies used

ER (SP1, Ventana 790-4324)
HER2 (4B5, Ventana 790-2991)
Ki67 (30-9, Ventana 790-4286)
Pan-Keratin (AE1/AE3/PCK26, Ventana 760-2135)
Vimentin (V9, Ventana 790-2917)
p53 (DO-7, Ventana 790-2912)
CK5/6 (D5/16B4, Ventana 790-4554)
CK8/18 (B22.1 & B23.1, Ventana 760-4344)
CD45 (RP2/18, Ventana 760-2505)
p63 (4A4, Ventana 790-4509)

	Synaptophysin (SP11, Ventana 790-4407)<br>E-cadherin (36, Ventana 790-4497)
Validation	Clinical grade antibodies.

Animals and other organisms

Policy information about studies involving animals; ARRIVE guidelines recommended for reporting animal research

Laboratory animals	5-7 week-old female NOD.Cg-PrkdcscidIl2rgtm1Sug/JicTac (NOG)(Taconic) or NOD.Cg-Prkdcscid Il2rgtm1Wjl/SzJ (NSG)(Jax).
Wild animals	Study did not involve wild animals.
Field-collected samples	Study did not involve samples collected from the field.
Ethics oversight	Facility Animal Care Committee at the Goodman Cancer Research Centre of McGill University (2014-7514)

Note that full information on the approval of the study protocol must also be provided in the manuscript.

Human research participants

Policy information about studies involving human research participants

Population characteristics	Patients with 1) ER-; 2) HER2+; 3) high-grade ER+; 4) metastatic; or 5) rare histological variants of breast cancer undergoing diagnosis and/or management at McGill University Health Centre or Jewish General Hospital, Montreal, QC, Canada.
Recruitment	Patients were identified based on inclusion criteria listed above. A clinical research assistant who is not involved in the care of these patients recruited patients after receiving permission from the most responsible physician.
Ethics oversight	All tissue was collected with informed consent under REB-approved protocols at the McGill University Health Centre and Jewish General Hospital.

Note that full information on the approval of the study protocol must also be provided in the manuscript.
